# The Business Side of Practice

**Published:** 1909-01-23

**Authors:** 


### The Business Side of Practice.

A well-known American financier has lately given it as his matured opinion that in a business sense physicians are a hopelessly irresponsible, unmathematical and careless class of society. This general accusation has been raised before now, in one shape or another, by candid critics of the medical profession on both sides of the Atlantic. British practitioners indeed are quite used to it, and can now listen, without a wince or a blush to reflections upon their "conspicuous want of business ability," and "their gross lack of method in dealing with professional affairs," which are still perhaps capable of goading their American brethren into angry denial or penitent reform. Whether the reproach is entirely merited or not, its frequent repetition seems to show that there is beneath it some solid foundation of fact. Nevertheless there are many medical men in every branch of private practice who take an intelligent practical interest in the financial side of their daily labours and keep their books in the most perfect order; and it is quite probable that their numbers have been underestimated. It must also be remembered that the practice of medicine cannot ever be conducted on the lines of a strictly commercial undertaking. It is one of the peculiar privileges of the profession to contribute very largely to the common good by unpaid, and often unappreciated, services; and that privilege is one which no high-minded member of it would care to see abolished. Mr. Rudyard Kipling recognised this in the striking address which he delivered to the Middlesex Hospital students last autumn.

Beyond that, however, there are many ways in which the bulk of medical men could, and in justice to their families and dependants ought to, improve the business side of their work. Because they labour gladly for Lazarus without hope of recompense, there is no reason why Dives should escape his just debt, in the same way. Medical men undergo a scientific training by which accuracy, organisation, and method are conspicuously inculcated. The standard of their preliminary education is as high as that of the members of the other professions, the Bar possibly excluded. Yet when a doctor dies it frequently happens that his widow and children lose large sums of outstanding book debts, uncollectable through negligent book-keeping. Possibly the profession is too much trammelled, as some believe, by traditions which have long been abandoned, if they ever existed, in other walks of life. The old custom of sending out bills but once a year or once in six months is one of these which has been thus attacked. It dies hard, partly because it is supposed to be the hall-mark of a high-class practice; but it is undoubtedly a source of avoidable loss in more ways than one. The practitioner loses interest on his gross annual receipts, and he also makes many bad debts through removals and through the curious impermanence of human gratitude. The precise intervals at which a medical man should send out his accounts must depend largely upon individual circumstances, and no hard and fast rule is possible. But this is a business age, and doctors in the long run will gain rather than lose the respect of their patients by conformity with the spirit of the times.

An American physician in a recent essay on this question points out how anomalous are the negligence and short-sightedness of medical men in their business affairs, when compared with their alertness to recognise and adopt scientific innovations and improvements of acknowledged value. Although this passion for novelty sometimes far outruns discretion, it is still something to the good that in bulk medical men do make such constant efforts to keep up with the march of progress; and it would be better still could this other reproach be removed also. Not that we advocate the importation of what is euphemistically called commercial morality into professional dealings. Commissions and illicit or under-hand dodges of all kinds are at present practically unknown among the medical practitioners of this country; though business men, arguing from their knowledge of human nature as met with in the course of business, not infrequently decline to believe this statement. If the adoption of system and method necessarily involved a risk of the introduction also of what is, in one word, dishonesty, we would far rather that doctors remained "unbusinesslike." But we have more faith in the profession at large than to believe that this is so.

